# Association of Serum Carotenoid Levels With N-Terminal Pro-Brain-Type Natriuretic Peptide: A Cross-Sectional Study in Japan

**DOI:** 10.2188/jea.JE20120087

**Published:** 2013-05-05

**Authors:** Koji Suzuki, Junichi Ishii, Fumihiko Kitagawa, Atsuhiro Kuno, Yasuhiro Kusuhara, Junichi Ochiai, Naohiro Ichino, Keisuke Osakabe, Keiko Sugimoto, Hiroya Yamada, Yoshinori Ito, Nobuyuki Hamajima, Takashi Inoue

**Affiliations:** 1 Department of Public Health, Fujita Health University School of Health Sciences, Toyoake, Aichi, Japan; 1 藤田保健衛生大学医療科学部公衆衛生学教室; 2 Department of Joint Research Laboratory of Clinical Medicine, Fujita Health University School of Medicine, Toyoake, Aichi, Japan; 2 藤田保健衛生大学医学部臨床検査部; 3 Department of Joint Research Laboratory of Clinical Medicine, Fujita Health University Hospital, Toyoake, Aichi, Japan; 3 藤田保健衛生大学病院臨床検査部; 4 Department of Medical Zoology, Fujita Health University School of Health Sciences, Toyoake, Aichi, Japan; 4 藤田保健衛生大学医療科学部医動物学教室; 5 Department of Medical Electronics, Fujita Health University School of Health Sciences, Toyoake, Aichi, Japan; 5 藤田保健衛生大学医療科学部医用工学教室; 6 Department of Clinical Physiology, Fujita Health University School of Health Sciences, Toyoake, Aichi, Japan; 6 藤田保健衛生大学医療科学部臨床生理検査学教室; 7 Department of Hygiene, Fujita Health University School of Medicine, Toyoake, Aichi, Japan; 7 藤田保健衛生大学医学部衛生学教室; 8 Department of Preventive Medicine, Nagoya University Graduate School of Medicine, Nagoya, Japan; 8 名古屋大学大学院医学系研究科予防医学教室

**Keywords:** carotenoids, N-terminal pro-brain-type natriuretic peptide, cross-sectional study

## Abstract

**Background:**

Several epidemiologic studies have reported an inverse association between serum levels of carotenoids and cardiovascular disease risk. However, no studies have reported an association between serum carotenoids and N-terminal pro-brain-type natriuretic peptide (NT-proBNP) in the general population.

**Methods:**

In this cross-sectional study, we investigated whether serum carotenoids were associated with serum NT-proBNP in 1056 Japanese subjects (390 men, 666 women) who attended a health examination. Serum levels of carotenoids were separately determined by high-performance liquid chromatography. Serum NT-proBNP level was measured by electrochemiluminescence immunoassay.

**Results:**

Serum NT-proBNP was elevated (≥55 pg/ml) in 31.8% of men and 48.2% of women. Multivariate logistic regression analyses adjusted for confounding factors showed a significant association between the highest quartile of serum α-carotene and elevated NT-proBNP in men (odds ratio [OR] = 0.40, 95% CI = 0.19–0.82, *P* for trend = 0.005) and women (OR = 0.62, 95% CI = 0.39–0.99, *P* for trend = 0.047). In women, moreover, elevated serum NT-proBNP was significantly associated with serum canthaxanthin (OR = 0.57, 95% CI = 0.36–0.90 for highest quartile, *P* for trend = 0.026) and β-cryptoxanthin (OR = 0.53, 95% CI = 0.32–0.85 for highest quartile, *P* for trend = 0.026), after adjusting for potential confounders.

**Conclusions:**

Higher levels of serum carotenoids were associated with lower risk of elevated serum NT-proBNP levels after adjusting for possible confounders, which suggests that a diet rich in carotenoids could help prevent cardiac overload in the Japanese population.

## Abstract (ja)

**【背景】:**

いくつかの観察的疫学研究は血清カロテノイドの高値が心血管疾患死亡率低下と関連することを示唆している。しかし一般住民を対象として血清カロテノイド値と心負荷マーカーである血清NT-proBNP値との関連について調査した研究はない。

**【方法】:**

日本人の住民健診受診者1056人（男390人、女666人）を対象として血清カロテノイド値とNT-proBNP値との関連について調査を行った。血清カロテノイド値は高速液体クロマトグラフィーにより分画測定し、血清NT-proBNP値は電気化学発光免疫測定法により測定した。

**【結果】:**

血清NT-proBNP値が55pg/ml以上の者の割合は、男で31.8％、女性で48.2％であった。血清カロテノイド値を性別に4等分し、交絡要因を調整した血清NT-proBNP高値（55pg/ml以上）のオッズ比を求めたところ、男女ともに血清*α*-カロテンの最高値群で有意に低いオッズ比を得た(男性：OR = 0.40、95%CI = 0.19-0.82、女性：OR = 0.62、95%CI = 0.39-0.99)。 さらに女性では血清カンタキサンチンとβ-クリプトキサンチンの最高値群もNT-proBNP高値のオッズ比は有意に低かった(カンタキサンチン最高値群：OR = 0.57、95%CI = 0.36-0.90、*β*-クリプトキサンチン最高値群：OR = 0.53、95%CI = 0.32-0.85)。

**【結論】:**

日本人住民健診受診者を対象として、*α*-カロテンなどのいくつかの血清カロテノイドの高値は血清NT-proBNP高値のリスク低下との関連を認めた。カロテノイドの豊富な食事の摂取は、心負荷リスクの軽減に関与する可能性が示唆された。

## INTRODUCTION

Brain-type natriuretic peptide (BNP) is secreted predominantly from the ventricular myocardium in response to increased ventricular wall stretch.^1^ In the process of BNP secretion, pro-BNP in cardiomyocytes is cleaved into the active hormone BNP and the inactive N-terminal pro-BNP (NT-proBNP). Circulating levels of these peptides are substantially increased in people with heart failure (HF) and during acute coronary syndromes and are established biomarkers for diagnosis, prognosis, and management in patients with HF and cardiovascular disease (CVD).^2^^–^^4^ Moreover, prospective epidemiologic studies have shown that elevated NT-proBNP is associated with increased CVD risk in the general population.^5^^–^^7^

Fruits and vegetables contain more than 40 carotenoids routinely absorbed and metabolized by humans.^8^ Circulating carotenoids are regarded as useful biomarkers of total dietary intake of vegetables and fruits.^9^ Epidemiologic studies found that serum, plasma, and adipose levels of various carotenoids were inversely associated with CVD risk.^10^^–^^12^ Carotenoids have various biologic functions, including antioxidant anti-inflammatory effects.^13^^,^^14^ Carotenoids may reduce CVD risk, in part because of their antioxidant and anti-inflammatory properties.^15^

Oxidative stress is associated with HF pathogenesis. Experimental and clinical studies have shown that generation of reactive oxygen species (ROS) is increased in HF.^16^^,^^17^ Several small case-control studies have reported that circulating levels of antioxidants, such as β-carotene and vitamin E, were lower in HF patients than in control subjects and that the reductions correlated with the severity of cardiac overload.^18^^,^^19^ Although some studies have reported associations between carotenoids and cardiovascular complications, no epidemiologic studies have evaluated the associations between serum levels of carotenoids and NT-proBNP, a biomarker of cardiac load. We investigated whether circulating carotenoids were independently associated with NT-proBNP in the Japanese general population.

## METHODS

Health examinations of inhabitants aged 39 years or older have been performed in Yakumo Town, Hokkaido Prefecture, Japan, since 1982.^20^ The initial population in the present study comprised 1178 adults (428 men, 750 women) who attended health examinations in August 2003 or August 2004. Among these 1178 adults, we excluded 28 who had serum samples that were inadequate for measurement of NT-proBNP or carotenoid levels and 94 with heart disease or kidney disease. Ultimately, data from 1056 subjects (390 men, 666 women) were analyzed. All study protocols were approved by the ethics committee of Fujita Health University (approval number 11-101).

Fasting serum samples were collected during health examinations, and sera were separated from blood cells by centrifugation within 1 hour. Serum samples were stored at −80°C until analyzed for carotenoids and NT-proBNP. Serum carotenoids and NT-proBNP levels were measured by the end of the year in which the sera were collected (2003 or 2004). NT-proBNP and carotenoids remain stable if the serum is frozen at −80°C until analyzed.^21^^,^^22^ Serum levels of carotenoids were separately determined by high-performance liquid chromatography.^23^ The coefficients of variation for the repeatability and reproducibility of the assay of carotenoids were 4.5% to 9.2% and 9.2% to 20.0%, respectively.^23^ Serum NT-proBNP level was measured by electrochemiluminescence immunoassay (Roche Diagnostics, Tokyo, Japan). The within-run coefficient of variation ranged from 1.3% to 2.4% and the between-run coefficient of variation ranged from 2.9% to 6.1% for the NT-proBNP assay.^24^ Other biochemical analyses of blood were performed using autoanalyzers in the laboratory at Yakumo General Hospital.

Trained nurses administered a questionnaire on health and daily lifestyle habits, smoking (current smoker, ex-smoker, or non-smoker), alcohol consumption (regular drinker, ex-drinker, or non-drinker), and history of major illness. Anthropometric indices (height, weight, and waist and hip circumferences) and blood pressure were measured during the health examination. Body mass index (BMI) was calculated as weight divided by the height squared (kg/m^2^). Estimated glomerular filtration rate (eGFR) was used to assess kidney function, according to the following equation: eGFR (ml/min/1.73 m^2^) = 194 × creatinine^−1.094^ × age^−0.287^ (× 0.739 if female).^25^

All statistical analyses were conducted using JMP version 9.0 software (SAS Institute, Cary, NC, USA). Because serum levels of carotenoids and NT-proBNP are distributed logarithmically, we used logarithms of these variables in the analyses. Categorical variables were compared by the chi-square test; continuous variables were analyzed using the *t*-test. Relationships between levels of serum carotenoids and NT-proBNP were evaluated by partial correlation analyses. Logistic regression analysis was used to estimate odds ratios (ORs) with 95% CIs for elevated NT-proBNP, adjusted for potential confounders. Elevated NT-proBNP was defined as a level of 55 pg/ml or higher, based on the findings of a previous report.^26^ Low serum levels of carotenoids were generally associated with male sex, young age, smoking, alcohol drinking, hyperglycemia, hypertension, high serum cholesterol, high serum γ-glutamyltransferase (γ-GTP), low kidney function, and obesity.^27^^–^^31^ We used age, BMI, smoking habit, alcohol consumption, eGFR, serum total cholesterol level, serum γ-GTP activity, hemoglobin (Hb) A1c, and systolic blood pressure (SBP) as potential confounders, based on the findings of earlier studies.^27^^–^^31^ A *P* value of less than 0.05 was considered statistically significant.

## RESULTS

Mean age was 61.9 years (range: 39–87 years) in men and 59.4 years (range: 39–86 years) in women. Serum NT-proBNP was significantly higher in women than in men. Serum levels of carotenoids, including zeaxanthin/lutein, canthaxanthin, β-cryptoxanthin, lycopene, α-carotene, and β-carotene, were all significantly higher in women than in men. Table 1 shows a comparison of the characteristics of subjects with elevated (≥55 pg/ml) and low serum NT-proBNP. The proportion of subjects with elevated serum NT-proBNP was 31.8% in men and 48.2% in women. Mean age and SBP were significantly higher in subjects with high NT-proBNP than in those with low NT-proBNP. Serum levels of β-cryptoxanthin and lycopene and eGFR were significantly lower in subjects with high NT-proBNP than in those with low NT-proBNP.

**Table 1.** Characteristics of study subjects

		Men		*P*	Women		*P*
	
NT-proBNP	(pg/ml)	<55	≧55		<55	≧55	
*n*		266 (68.2%)	124 (31.8%)		345 (51.8%)	321 (48.2%)	
Age	(y)^a^	59.3 ± 10.4	67.5 ± 8.5	<0.001^b^	57.2 ± 9.6	61.7 ± 10.6	<0.001^b^
NT-proBNP	(pg/ml)^c^	22.3 (15.3–37.3)	131.1 (71.5–180.6)	<0.001^b^	29.4 (22.3–43.3)	100.5 (68.0–128.1)	<0.001^b^
Zeaxanthin/lutein	(µmol/l)^c^	1.26 (0.88–1.60)	1.24 (0.92–1.64)	0.746^b^	1.47 (1.06–1.81)	1.45 (1.00–1.85)	0.743^b^
Canthaxanthin	(µmol/l)^c^	0.015 (0.011–0.023)	0.013 (0.010–0.021)	0.068^b^	0.020 (0.016–0.030)	0.018 (0.014–0.025)	0.002^b^
β-Cryptoxanthin	(µmol/l)^c^	0.18 (0.11–0.30)	0.16 (0.11–0.26)	0.248^b^	0.32 (0.22–0.50)	0.31 (0.21–0.44)	0.200^b^
Lycopene	(µmol/l)^c^	0.37 (0.23–0.64)	0.29 (0.19–0.50)	0.006^b^	0.54 (0.36–0.87)	0.47 (0.31–0.74)	0.012^b^
α-Carotene	(µmol/l)^c^	0.087 (0.056–0.145)	0.074 (0.048–0.114)	0.044^b^	0.14 (0.10–0.21)	0.13 (0.09–0.19)	0.049^b^
β-Carotene	(µmol/l)^c^	0.44 (0.25–0.76)	0.42 (0.26–0.79)	0.728^b^	0.93 (0.61–1.48)	0.90 (0.61–1.40)	0.525^b^
Total cholesterol	(mg/dl)^a^	207.8 ± 32.2	202.9 ± 32.5	0.166^b^	222.4 ± 34.3	212.6 ± 30.6	<0.001^b^
HDL-cholesterol	(mg/dl)^a^	53.1 ± 12.0	53.2 ± 13.7	0.979^b^	61.7 ± 13.2	60.3 ± 13.0	0.165^b^
AST	(IU/l)^c^	25 (20–29)	26 (21–29)	0.309^b^	22 (18–26)	23 (19–27)	0.728^b^
ALT	(IU/l)^c^	27 (20–34)	24 (19–30)	0.077^b^	22 (15–29)	19 (15–24)	0.728^b^
γ-GTP	(IU/l)^c^	33 (20–51)	36 (19–57)	0.312^b^	18 (11–26)	17 (11–24)	0.055^b^
BMI	(kg/m^2^)^a^	24.1 ± 3.1	24.2 ± 3.3	0.884^b^	24.0 ± 3.3	23.6 ± 3.6	0.089^b^
SBP	(mm Hg)^a^	135.2 ± 18.4	143.8 ± 20.8	<0.001^b^	131.2 ± 17.6	137.8 ± 20.6	<0.001^b^
DBP	(mm Hg)^a^	87.2 ± 10.9	89.1 ± 12.7	0.125^b^	83.5 ± 10.2	84.8 ± 11.7	0.124^b^
Hemoglobin A1c	(%)^a^	5.3 ± 0.7	5.4 ± 0.7	0.186^b^	5.3 ± 0.7	5.2 ± 0.6	0.232^b^
eGFR	(ml/min/1.73 m^2^)^a^	81.7 ± 17.3	72.7 ± 16.4	<0.001^b^	81.7 ± 16.1	77.6 ± 15.3	<0.001^b^
Smoking habit	Current smoker	90 (33.8%)	36 (29.3%)	0.390^d^	41 (12.0%)	32 (10.0%)	0.252^d^
	Ex-smoker	110 (41.4%)	60 (48.8%)		35 (10.2%)	23 (7.2%)	
	Nonsmoker	66 (24.8%)	27 (22.0%)		267 (77.8%)	264 (82.8%)	
Drinking habit	Current drinker	156 (58.9%)	73 (58.9%)	0.012^d^	51 (14.8%)	54 (16.9%)	0.061^d^
	Ex-drinker	11 (4.2%)	15 (12.1%)		12 (3.5%)	26 (0.9%)	
	Nondrinker	98 (36.9%)	36 (29.0%)		281 (81.7%)	263 (82.2%)	

Abbreviations: NT-proBNP, N-terminal pro-brain-type natriuretic peptide; HDL, high-density lipoprotein; AST, aspartate aminotransferase; ALT, alanine aminotransferase; γ-GTP, γ-glutamyl transpeptidase; BMI, body mass index; SBP, systolic blood pressure; DBP, diastolic blood pressure; eGFR, estimated glomerular filtration rate.

^a^Data are shown as mean (SD).

^b^*t*-test.

^c^Data are shown as geometric mean (25th–75th percentiles).

^d^χ^2^ test.

The multivariable-adjusted partial correlation coefficients between serum NT-proBNP level and serum levels of carotenoids are shown in Table 2. Serum NT-proBNP was significantly inversely associated with serum levels of canthaxanthin, lycopene, and α-carotene among women. No significant associations between serum NT-proBNP and serum levels of carotenoids were observed among men after adjustment for potential confounders.

**Table 2.** Relationships of serum NT-proBNP with serum retinol and serum carotenoids by sex

Variable	Men				Women			
	
	Age-adjusted	*P*	Multivariable- adjusted^a^	*P*	Age-adjusted	*P*	Multivariable- adjusted^a^	*P*
Ln Zeaxanthin/lutein	0.018	0.724	−0.063	0.206	0.034	0.388	−0.022	0.779
Ln Canthaxanthin	−0.101	0.045	−0.068	0.198	−0.157	<0.001	−0.131	0.007
Ln β-Cryptoxanthin	−0.024	0.631	−0.084	0.111	−0.017	0.667	−0.036	0.649
Ln Lycopene	−0.098	0.053	−0.014	0.776	−0.144	<0.001	−0.090	0.053
Ln α-Carotene	−0.051	0.320	−0.019	0.707	−0.081	0.038	−0.100	0.029
Ln β-Carotene	0.040	0.429	−0.023	0.599	−0.016	0.675	−0.078	0.186

Abbreviation: NT-proBNP, N-terminal pro-brain-type natriuretic peptide.

^a^Adjusted for age, body mass index, smoking habit, drinking habit, estimated glomerular filtration rate, total cholesterol, γ-glutamyl transpeptidase, hemoglobin A1c, and systolic blood pressure.

We calculated the OR and 95% CI for elevated serum NT-proBNP, adjusted for confounding factors, by quartile of serum carotenoids (Table 3). In women, the OR for elevated serum NT-proBNP was significantly lower in the highest quartiles of serum canthaxanthin, β-cryptoxanthin, and α-carotene levels than in the lowest quartile. Among men, the OR was significantly lower in the highest and third quartiles of serum α-carotene levels as compared with the lowest quartile. Moreover, ORs for elevated serum NT-proBNP tended to decrease as serum canthaxanthin and β-cryptoxanthin increased in men (*P* for trend = 0.054 and 0.059, respectively).

**Table 3.** Adjusted^a^ odds ratios for elevated serum NT-proBNP according to serum carotenoid levels

	Quartile of serum carotenoid level				*P* for trend

	1st	2nd	3rd	4th	
Men					
Zeaxanthin/lutein	1.00	1.08 (0.54–2.15)	0.70 (0.35–1.40)	0.80 (0.39–1.64)	0.332
Canthaxanthin	1.00	0.90 (0.47–1.70)	0.46 (0.22–0.94)	0.61 (0.31–1.18)	0.054
β-Cryptoxanthin	1.00	1.16 (0.59–2.29)	0.78 (0.39–1.57)	0.54 (0.26–1.13)	0.059
Lycopene	1.00	1.26 (0.66–2.43)	1.16 (0.60–2.27)	0.55 (0.26–1.13)	0.141
α-Carotene	1.00	0.84 (0.43–1.63)	0.47 (0.23–0.95)	0.40 (0.19–0.82)	0.005
β-Carotene	1.00	1.13 (0.57–2.28)	0.56 (0.27–1.15)	0.79 (0.38–1.64)	0.238
Women					
Zeaxanthin/lutein	1.00	0.73 (0.46–1.17)	0.74 (0.46–1.17)	0.65 (0.40–1.05)	0.101
Canthaxanthin	1.00	1.08 (0.67–1.73)	0.97 (0.63–1.51)	0.57 (0.36–0.90)	0.026
β-Cryptoxanthin	1.00	0.86 (0.54–1.37)	1.03 (0.64–1.63)	0.53 (0.32–0.85)	0.026
Lycopene	1.00	0.90 (0.57–1.42)	1.00 (0.63–1.59)	0.67 (0.41–1.08)	0.165
α-Carotene	1.00	0.89 (0.56–1.40)	0.79 (0.50–1.26)	0.62 (0.39–0.99)	0.047
β-Carotene	1.00	0.87 (0.55–1.39)	0.84 (0.52–1.35)	0.67 (0.41–1.08)	0.108

Abbreviation: NT-proBNP, N-terminal pro-brain-type natriuretic peptide.

^a^Adjusted for age, body mass index, smoking habit, drinking habit, estimated glomerular filtration rate, total cholesterol, γ-glutamyl transpeptidase, hemoglobin A1c, and systolic blood pressure.

Data are expressed as odds ratio (95% CI).

Elevated NT-proBNP was defined as a level ≥55 pg/ml.

## DISCUSSION

Serum levels of several carotenoids were inversely associated with serum NT-proBNP level in Japanese women, even after adjusting for possible confounding factors. Weak inverse associations were seen in Japanese men. These results suggest that high intake of vegetables and fruits ameliorates cardiac overload.

We observed no association between serum α-carotene and serum NT-proBNP in men (Table 2). However, the OR for elevated serum NT-proBNP decreased across increasing quartiles of serum α-carotene (Table 3). This discrepancy may be due to the difference in statistical methods. Partial correlation represents the strength of the relationship between 2 continuous variables after controlling for other variables. OR represents the strength of an association between 2 binary valuables. Although serum α-carotene was not linearly related with serum NT-proBNP, higher serum α-carotene was associated with a lower risk of elevated NT-proBNP (≥55 pg/ml) in men.

Elevated BNP and NT-proBNP levels are strongly associated with HF and CVD.^2^^–^^4^^,^^32^^,^^33^ BNP is predominantly produced in cardiac tissue,^1^ and the left ventricle is the primary source of circulating BNP in the normal state and under conditions of left ventricular dysfunction.^32^^,^^33^ The BNP gene is activated in cardiomyocytes in response to increased stress of the myocardial wall. Thus, precursor proBNP is produced intracellularly and then cleaved by endoprotease upon secretion, which results in the formation of biologically inert NT-proBNP and biologically active BNP.^34^ BNP assists in regulating blood pressure, blood volume, and sodium balance.^35^ Prospective studies have shown that elevated NT-proBNP is a predictor of total CVD and coronary heart disease (CHD) mortality, even after controlling for traditional cardiovascular risk factors.^5^^–^^7^

Increased oxidative stress contributes to HF pathogenesis. Clinical studies have reported that chronic HF was associated with increased plasma oxidants, such as malondialdehyde and lipid peroxides, and decreased plasma levels of antioxidant vitamins, including vitamins C and E and β-carotene.^18^^,^^19^^,^^36^ A large prospective study found that plasma vitamin C was inversely associated with HF risk in a healthy European population.^37^

ROS are highly reactive molecules that can oxidize DNA, proteins, and lipids. They are produced by several mechanisms such as mitochondrial electron transport, NAD(P)H oxidase, and xanthine oxidase within cardiac myocytes. ROS cause contractile failure and structural damage in the myocardium.^38^ Oxidative stress is caused by an imbalance between ROS generation and the antioxidant defense system. Increased oxidative stress also indicates enhanced ROS production. Therefore, decreased circulating levels of antioxidants such as vitamin C and carotenoids may increase ROS.

Carotenoids are potent quenchers of singlet molecular oxygen^14^ and, by quenching free radicals, have an important role in decreasing activation of proinflammatory pathways.^13^ Dietary intake of carotenoids was found to be associated with decreased CVD risk.^15^^,^^39^^,^^40^ Plasma concentrations of carotenoids are believed to be useful biomarkers of total dietary intake of fruits and vegetables.^9^ Several prospective epidemiologic studies have shown that high serum levels of carotenoids are associated with low CHD risk.^10^^,^^12^^,^^41^ A cohort study by Morris et al^12^ found that high serum levels of total carotenoids were associated with lower risk of incident CHD in men. The Basel Prospective Study^41^ found that the risk of ischemic heart disease and stroke was significantly increased in subjects with initially low plasma levels of α- and β-carotene. In addition, a Japanese prospective study reported that high serum levels of α- and β-carotene were significantly associated with low CVD risk.^10^ Antioxidant and anti-inflammatory properties may explain the possible role of carotenoids in HF and CVD prevention.

Several limitations of this study warrant mention. First, although some epidemiologic studies have reported an inverse association of circulating levels of carotenoids with CVD risk,^10^^,^^12^^,^^41^ large randomized trials have found β-carotene supplementation to be ineffective in preventing CVD.^42^^,^^43^ Because high doses of carotenoids could have a pro-oxidant effect, it is possible that the effects of carotenoids in protecting against CVD disappear at the high doses used in supplementation studies. Supplementation with β-carotene had little effect on our results, because only 3 men and 9 women in this study used β-carotene supplements. Second, NT-proBNP level depends on sex, age, and physiologic conditions such as renal function.^44^^–^^46^ We conducted sex-specific analyses because women had NT-proBNP levels that were approximately 1.4 times those of men in this study, as was the case in previous studies.^44^^,^^45^ The age-related increase in NT-proBNP may reflect the higher prevalence of subclinical cardiac disease and renal dysfunction in older subjects.^47^ Because NT-proBNP is mainly cleared by the kidneys, circulating levels are often elevated in patients with renal dysfunction. The possibility of residual confounding cannot be completely excluded, although other confounding variables were appropriately adjusted for in our analyses. Finally, this study was unable to examine issues of causality, due to the cross-sectional design. A prospective study is thus required to confirm our results and clarify causal relationships.

In conclusion, levels of serum carotenoids, which are markers of fruit and vegetable intake, were inversely associated with risk of elevated serum NT-proBNP in the general Japanese population, although serum levels of all carotenoids were not linearly related to serum NT-proBNP. These findings suggest that a diet rich in vegetables and fruits prevents cardiac overload and thus reduces CVD risk.

## ONLINE ONLY MATERIALS

Abstract in Japanese.
