# Supplementary material for: Association of Serum Carotenoid Levels With N-Terminal Pro-Brain-Type Natriuretic Peptide: A Cross-Sectional Study in Japan
Source: J Epidemiol. 2013 May 5;23(3):163–8. doi: 10.2188/jea.JE20120087 (PMC3700260; doi:10.2188/jea.JE20120087)
Supplement: Abstract in Japanese. [file je-23-163-s001.pdf]

日本人集団における血清カロテノイドとN-terminal pro-brain-type natriuretic peptide（NT-proBNP）との関連

鈴木康司[1], 石井潤一[2], 北川文彦[3], 久野 貴弘[3], 楠原康弘[4], 落合潤一[5], 市野直浩[6], 刑部恵介[6], 杉本恵子[6], 山田宏哉[7], 伊藤宜則[8], 浜島信之[8], 井上孝[1]

1 藤田保健衛生大学医療科学部公衆衛生学教室
2 藤田保健衛生大学医学部臨床検査部
3 藤田保健衛生大学病院臨床検査部
4 藤田保健衛生大学医療科学部医動物学教室
5 藤田保健衛生大学医療科学部医用工学教室
6 藤田保健衛生大学医療科学部臨床生理検査学教室
7 藤田保健衛生大学医学部衛生学教室
8 名古屋大学大学院医学系研究科予防医学教室

【背景】いくつかの観察的疫学研究は血清カロテノイドの高値が心血管疾患死亡率低下と関連することを示唆している。しかし一般住民を対象として血清カロテノイド値と心負荷マーカーである血清 NT-proBNP 値との関連について調査した研究はない。

【方法】日本人の住民健診受診者 1056 人（男 390 人、女 666 人）を対象として血清カロテノイド値と NT-proBNP 値との関連について調査を行った。血清カロテノイド値は高速液体クロマトグラフィーにより分画測定し、血清 NT-proBNP 値は電気化学発光免疫測定法により測定した。

【結果】血清 NT-proBNP 値が 55pg/ml 以上の者の割合は、男で 31.8%、女性で 48.2%であった。血清カロテノイド値を性別に 4 等分し、交絡要因を調整した血清 NT-proBNP 高値（55pg/ml 以上）のオッズ比を求めたところ、男女ともに血清α-カロテンの最高値群で有意に低いオッズ比を得た(男性：OR = 0.40、95%CI = 0.19-0.82、女性：OR = 0.62、95%CI = 0.39-0.99)。 さらに女性では血清カンタキサンチンとβ-クリプトキサンチンの最高値群も NT-proBNP 高値のオッズ比は有意に低かった(カンタキサンチン最高値群：OR = 0.57、95%CI = 0.36-0.90、β-クリプトキサンチン最高値群：OR = 0.53、95%CI = 0.32-0.85)。

【結論】日本人住民健診受診者を対象として、α-カロテンなどのいくつかの血清カロテノイドの高値は血清 NT-proBNP 高値のリスク低下との関連を認めた。カロテノイドの豊富な食事の摂取は、心負荷リスクの軽減に関与する可能性が示唆された。

キーワード：カロテノイド、NT-proBNP、横断研究
